# Characteristics of cell adhesion molecules expression and environmental adaptation in yak lung tissue

**DOI:** 10.1038/s41598-025-95882-2

**Published:** 2025-03-29

**Authors:** Huizhen Wang, Nating Huang, Minglu Tan, Xun Zhang, Jiarui Chen, Qing Wei

**Affiliations:** 1 College of Eco-Environmental Engineering, Qinghai University, Xining, Qinghai China; 2 Livestock and Veterinary Station of Huangyuan County, Xining, Qinghai China; 3 State Key Laboratory of Plateau Ecology and Agriculture, Qinghai University, Xining, Qinghai China

**Keywords:** Yak, Lung tissue, Cell adhesion molecules (CAMs), Plateau environmental adaptation, Animal physiology, Cell adhesion

## Abstract

Cell Adhesion Molecules (CAMs) play a crucial role in regulating immune responses and repairing damage caused by hypoxia. However, the relationship between the expression characteristics of CAMs in yak lung tissues and their adaptation to the plateau environment remains unclear. To address this question, we compared lung tissues from yaks and cattle at the same altitude. After digesting the lung tissues with trypsin or Type I collagenase for varying durations, we observed that fewer cells were isolated from yak tissues compared to cattle. RNA sequencing (RNA-seq) analysis revealed that the Differentially Expressed Genes (DEGs) in lung tissues of yaks and cattle were significantly enriched in cell adhesion-related pathways. Quantitative real-time PCR (qRT-PCR) further identified changes in the expression levels of five distinct types of CAMs. Among these, the cadherin family (*CDH1*,* CDH2*,* CDH11*,* PCDH12*,* CD34*) exhibited significantly higher expression in yaks than in cattle. These cadherins play a critical role in regulating lung inflammation and maintaining the alveolar-capillary barrier, thereby ensuring the structural stability of the lungs. Immunohistochemical staining demonstrated that the expression patterns of cell adhesion-related proteins (CDH1, CDH11, ITGB6, SELP, CD44) were largely consistent with the qRT-PCR results. In conclusion, compared to cattle, the enhanced cell adhesion capacity of yak lung tissues contributes to their superior adaptation to the harsh plateau environment.

## Introduction

The Qinghai-Tibet Plateau is a unique geographical unit that serves as a vital ecological security barrier for China and Asia^1^. This plateau is characterized by low partial pressure of oxygen, low ambient temperatures, and intense ultraviolet radiation, which pose serious challenges to the survival of both humans and animals inhabiting the area^2^. The indigenous animals of the plateau have developed excellent adaptations to the high-altitude environment through a long evolutionary and adaptive process^3^. For instance, the plateau pika (*Ochotona curzoniae*), a small mammal endemic to the Tibetan Plateau, enhances anaerobic glycolysis in somatic cells by upregulating the expression of LDH-C_4_ under hypoxic conditions, thereby reducing its reliance on oxygen and improving its ability to thrive in hypoxic environments^4^. Similarly, the Tibetan antelope (*Pantholops hodgsonii*) significantly enhances oxygen acquisition and storage under hypoxic conditions by increasing the expression of myoglobin in cardiac and skeletal muscles^5^. Among these indigenous species, the yak (*Bos grunniens*), as the only bovine species in the genus *Bos* that breeds in the harsh climatic conditions of the Qinghai-Tibet Plateau, has long been settled and widely distributed there^6^. Over long periods, yaks have evolved specialized mechanisms to adapt to the hypoxic and cold environments of the plateau through prolonged natural selection, ensuring their survival and reproduction in these extreme conditions^7^. As a result, the yak has become an ideal model species for researchers studying the environmental adaptations of plateau indigenous animals.

The lung is a critical organ for gas exchange between mammals and the external environment, and lung tissue is among the first to exhibit compensatory responses under hypoxic conditions on the plateau. Yak lung tissue has evolved a series of adaptive changes to cope with the plateau environment, including shorter and wider bronchi, well-developed respiratory muscles, larger alveolar surface areas, thicker alveolar septa, and thinner air-blood barriers^8,9^. Studies have shown that many physiological functions of lung tissue are linked to the expression of CAMs. CAMs are cell-surface glycoproteins that mediate physical interactions between neighboring cells and between cells and the extracellular matrix (ECM)^10^, thereby maintaining the structural stability of tissues. Based on their structural differences, CAMs can be classified into five categories: the cadherin family, the immunoglobulin superfamily, the selectin family, the integrin family, and other adhesion molecules^11,12^. Research has revealed a connection between CAMs and adaptation to hypoxia. HIFs significantly enhance the adhesion of cancer cells and vascular endothelial cells through pathways mediated by selectins and integrins^13^. Hypoxia influences integrin-mediated adhesion of mast cells to fibronectin, thereby alleviating inflammation^14^. Additionally, hypoxia upregulates the expression of VCAM-1, enhancing the adhesion of sickle red blood cells to aortic endothelial cells and contributing to vascular injury repair^15^. The upregulation of adhesion molecules under hypoxic conditions helps cells maintain a favorable environment for recovery at injury sites^16^. CEACAM-1, a member of the immunoglobulin superfamily, is considered as an angiogenic factor^17^. In a study using mouse and rat models to simulate chronic hypoxic conditions, hypoxia-preconditioned animals exhibited increased expression of CEACAM-1, suggests that CAMs, such as CEACAM-1, promote angiogenesis under hypoxic conditions, thereby helping animals avoid ischemic injuries and protecting tissues^18^.

In summary, there appears to be a correlation between CAMs and the adaptation of animals to the plateau environment. However, studies on the expression characteristics of CAMs in yak lung tissues and their relationship to adaptation to the plateau environment remain limited. Therefore, we selected lung tissues from yaks and cattle at the same altitude for this study. By comparing and analyzing the differences in cell adhesion capacity and CAMs expression in the lung tissues of yaks and cattle, we explored the significance of CAMs-specific expression in yak lung tissues for hypoxic adaptation. This study provides a theoretical foundation for further research on the mechanisms underlying yak adaptation to plateau environment.

## Materials and methods

### Experimental animals and treatments

In this study, three healthy adult male plateau-type yaks (XH-Y) and three healthy adult male cattle (XH-C), totaling six animals, were selected from Xunhua County, Qinghai Province (altitude 2,600 m). At the same altitude, yaks are a bovine species endemic to the Tibetan Plateau and serve as the experimental group, while cattle are a common bovine species and serve as the control group. The selected animals were all approximately 3–5 years old, as individuals in this age range have reached physiological maturity, with fully developed lung tissues and a high degree of adaptation to the plateau environment, ensuring an accurate representation of lung tissue characteristics. Yaks and cattle are killed by bloodletting in local slaughterhouse. Fresh tissues from the left lung diaphragmatic lobe of yaks and cattle were collected within 30 min of death. One portion of the tissues was placed in cryotubes after removing the fascia and stored in liquid nitrogen and brought back for spare, while the other portion was cut into small pieces of approximately 1 cm^3^ and fixed in 4% paraformaldehyde solution. All lung tissue samples were collected locally in Xunhua County, Qinghai Province, China, and not purchased. Animal experiments in this study were approved by the Animal Welfare and Ethics Committee of Qinghai University, and conducted in full compliance with the Guide for the Care and Use of Laboratory Animals, and adhered to the ARRIVE Guidelines. All methods were performed in accordance with the relevant guidelines and regulations.

### Cell digestion experiment

Yak lung tissue was placed in a sterile Petri dish containing PBS with double antibodies, and the tissue was minced into pieces approximately 1 mm^3^ in size after removing the fascia. Subsequently, 2–3 mL of the minced tissue mixture was transferred to a 15 mL centrifuge tube, and 4–5 times its volume of either 0.25% trypsin or 2 mg/mL Type I collagenase was added for digestion at 37 °C. These two enzyme concentrations represent the minimum concentrations required to effectively dissociate the tissue. During digestion, shake every 10 min to observe the state of digestion. At 1 h, 2 h, and 3 h of tissue digestion, the filtrate was collected, and the digestion was terminated by adding an equal volume of culture solution to the cell filtrate. Subsequently, the mixture was centrifuged at 1500 r for 5 min, the supernatant was discarded, and 3 mL of erythrocyte lysate was added and mixed thoroughly. After standing for 10 min, the mixture was centrifuged again under the same conditions, and the resulting white precipitate represented the target cell mass. The supernatant was discarded, and 3 mL of culture solution was added, then the pipette was used to gently blow and mix the solution to ensure the cells were dispersed as single cells. Finally, 3 mL of the cell suspension was transferred into cell culture flasks. The cells were observed and photographed under an inverted fluorescence microscope for counting. After completion, the cell culture flasks were placed in a constant-temperature incubator at 37 ℃ with 5% CO_2_ for further cultivation.

The cell digestion method employed in this study utilizes the enzymatic properties to disrupt cellular connections in lung tissue. Collagenase and trypsin are two enzymes with distinct mechanisms of action. Collagenase effectively hydrolyzes proteins, polysaccharides, and lipids within the ECM of connective and epithelial tissues, making it more suitable for tissue digestion^19,20^. Trypsin degrades cell surface proteins, leading to cell lysis and the release of intracellular DNA^21^. The lung tissues of yaks and cattle differ significantly in structure and composition. Yak lung tissue is rich in collagen fibers, with a more stable and elastic basement membrane structure, whereas cattle lung tissue is softer and structurally simpler^22^. Therefore, the use of Type I collagenase to digest yak lung tissues can effectively dissociate the tissues, while the use of trypsin to digest cattle lung tissues may yield higher cell viability and recovery rates. However, differences in tissue structure lead to variations in digestion times between the two species.

### RNA-seq and data analysis

Lung tissue samples collected from yaks and cattle were sent to Guangzhou Kidio Biotechnology Co. for RNA-seq. The study employed the Illumina paired-end sequencing method, which offers several advantages, including improved accuracy in data mapping, enhanced genome assembly quality, and increased capability for detecting structural variants^23^. RNA-seq primarily involves several key steps: extracting mRNA, reverse transcription, constructing sequencing libraries on the machine, synthesizing cDNA’s 1st and 2nd strand, building the cDNA libraries, and sequencing on the machine. For detailed methodology, please refer to the eukaryotic transcriptome experimental methods published on the Guangzhou Kidio Biotechnology Co., Ltd. Website (https://www.genedenovo.com/). We finally obtained the cDNA libraries and sequencing results of yak and cattle lung tissues. After sequencing, data analysis involves quality control and filtering of raw data, alignment to reference genomes, quantification, differential expression analysis, and gene annotation, among other steps. The reference genome version was BosGruv2.0 (http://ftp.ensembl.org/pub/release-110/fasta/bos_mutus/), and we used |Fold Change| ≥ 2 and FDR < 0.05 as the screening criteria for DEGs. The screened DEGs were functionally analyzed using the GO database (http://www.geneontology.org/) and the KEGG Pathway^24^ database (https://www.genome.jp/kegg/) to uncover their potential biological functions and pathways.

### Measurement of ion concentrations related to cell adhesion

The freeze-drying method preserves samples by freezing water-containing cells or tissues into a solid state at low temperatures and removing water through vacuum sublimation, thereby maximizing the retention of cellular structure and active components^25^. Approximately 10 g of lung tissue from yaks and cattle was freeze-dried using this method to obtain freeze-dried lung tissue. The freeze-dried tissue samples were then pulverized using a grinder. Accurately weigh 0.1 g of the lung tissue dry powder sample using a precision balance, and place it in a dedicated microwave digestion tank. First, 7 mL of HNO₃ was added to the digestion vessel, followed by 2 mL of H_2_O_2_. The combination of microwave heating and strong acids rapidly and thoroughly decomposes the biological tissue, releasing the metal ions within. Leave it overnight for adequate pre-dissolution, followed by microwave dissolution. Following digestion, allow it to cool and then place it in an acid drive unit with the temperature set at 170 ℃ for approximately 130 min to remove excess acid. Once the acid drive is complete, rinse the microwave digestion tank and lid with ultrapure water, and make up to 50 mL in a volumetric flask for spare. The sample solution was diluted 1,000-fold, and the calcium and magnesium ion concentrations were determined using inductively coupled plasma mass spectrometer (ICP-MS).

Ca²⁺ and Mg²⁺ are key regulators of cell adhesion^26^. Ca²⁺ regulates cell adhesion by mediating calcium transients, which promote the recruitment of connexins and the reorganization of the cytoskeleton^27^. On the other hand, Mg²⁺ modulates cell adhesion by binding to integrins and inducing conformational changes that alter their binding affinity to ligands, thereby influencing cell adhesion^28^. Therefore, we examined changes in Ca²⁺ and Mg²⁺ concentrations to evaluate the functional state of cell adhesion.

### qRT-PCR

Retrieve the CDS sequences of cell adhesion-related genes, such as *CDH1*,* CDH2*, and *CDH11*, for yaks and cattle from the NCBI database. Specific degenerate primers for these cell adhesion-related genes in yaks and cattle were designed using Oligo 7.0 software, and sent to Sangon Biotech (Shanghai) Co., Ltd. for synthesis. The primer sequences are listed in Table 1. qRT-PCR is a widely used technique for the quantitative analysis of gene expression, and the specificity of primers is critical to ensure accurate detection of target gene^29^. Our sources of primers include those reported in the literature and those designed in-house. All primers were validated before use, and the fluorescence quantitative PCR curves were single-peaked and stable with good experimental results. The primers were specific and effective.

**Table 1:** Primer sequences for target genes.

Primer name	Type	Sequence (5′→3′)
*CDH1*	Forward primer	CTGAGAACGAGGCTAATGTGGC
	Reverse primer	TGACGGTGGCTGTGGAAGTG
*CDH2*	Forward primer	ACGTTCTATGGTGAAGTCCCTGA
	Reverse primer	CTGATTCTGTAGATGGCGTTCC
*CDH11*	Forward primer	GTCAACGACAACCCACCAAAG
	Reverse primer	TCCATCCCATCTCCATCAACAA
*PCDH12*	Forward primer	GCCTCCTGTTCAGCAAATCTCC
	Reverse primer	AGGAGGTTCGACAGCTCTTCTTC
*CD34*	Forward primer	CGAAGACCCTTATTACGTGGAGAAC
	Reverse primer	GATTCACAATGGCCTTTCCCT
*SIGLEC1*	Forward primer	ACCGGAGTCGTGTACTACAGTTGC
	Reverse primer	GACAGGAGGTGAGACCGAAGAG
*ICAM3*	Forward primer	CAGGAAGCTCCAGACCTATGCC
	Reverse primer	CGTGGCTCACTACTGTGGCATT
*ALCAM*	Forward primer	AGGACAGCCTGAAGGAATTAGAAG
	Reverse primer	AGTGAACTGTGATGGCTGCTGA
*SELP*	Forward primer	AGTGTGAATATGTCAGAGAGTGCGG
	Reverse primer	AAGCCAAACACTCCAGCTCACT
*SELL*	Forward primer	GGAGCCCAACAACAGGAAGAGT
	Reverse primer	TGCATGACCAGGGTTTACAAGA
*SELPLG*	Forward primer	CCCAGAAGACGATGAAGACTATGA
	Reverse primer	GGACACCTCCAGAATGAATGACT
*ITGA3*	Forward primer	GGCAGACCTACCACAACGAGAT
	Reverse primer	TTCAGACAAATCCCAGTCCTTCC
*ITGA5*	Forward primer	AAGAATCTCAACAACTCGCAAAGC
	Reverse primer	GCCAGTCGCTCATCGGAAATA
*ITGB6*	Forward primer	GAGCACCGAGTCCTGCAAAGA
	Reverse primer	CAGTCACAGTCGCCATTATCTCC
*CD44*	Forward primer	ACTAACCCAGAAGACATCAACCC
	Reverse primer	TAGTCTCTGGTATCCGAGGTATTCT
*ESAM*	Forward primer	TGTTCCTGGGGCTGACTACTCT
	Reverse primer	CATGCCCGTTGTGACCTGAC
*VCAM1*	Forward primer	GCTCAGTTAGAGGATGCGGG
	Reverse primer	GCATTAGCAGACTTTCTGTGCT
*CEACAM1*	Forward primer	TACTAGAGGGGCAAGTGACCAG
	Reverse primer	GTTTCTGTATTTGTTGGAGATGGG
*ITGAV*	Forward primer	CACTGGAGGACTGAGATGAAGCA
	Reverse primer	CAATGCTGAATCCTCCTTGACAA
*JAM3*	Forward primer	GTCTCTGAAGATCTGGAACGTGAC
	Reverse primer	TGGCACGTCATTGCGGTAC
*GAPDH*	Forward primer	GGTCACCAGGGCTGCTTTTA
	Reverse primer	CCAGCATCACCCCACTTGAT

Approximately 0.1 g of lung tissue from yaks and cattle was weighed, and total RNA was extracted from these tissues following the instructions provided with the Total RNA Extraction Kit. The RNA concentration and OD values were measured using an ultramicro spectrophotometer (NanoDrop 2000). In this method, the instrument blank was zeroed, and 2.5 µL of the RNA solution was placed on the detection pedestal. The sample arm was lowered, and the absorbance values were measured using the accompanying software. The calculation formulas of RNA concentration and OD values are shown in Eqs. (1) and (2).

1 $$\:C=\frac{A}{ \varepsilon \:b}$$

Where *C* is the sample concentration (µg/mL), *A* is the absorbance value, *ε* is the wavelength-dependent molar extinction coefficient (ng cm/mL), and *b* is the optical path length (cm).

2 $$\:OD=\frac{{A}_{260}}{{A}_{280}}$$

Where *OD* represents the purity, *A*_*260*_ is the absorbance value at 260 nm with a 10 mm optical path length, and *A*_*280*_ is the absorbance value at 280 nm with a 10 mm optical path length. Additionally, $\:\frac{{A}_{260}}{{A}_{280}}$ > 1.8.

Subsequently, 1.2 µg of RNA was used for reverse transcription following the instructions provided with the Reverse Transcription Kit. The reverse transcription program was as follows: 94 ℃ for 15 min, 42 ℃ for 3 min, and 4 ℃ ∞. The resulting cDNA was stored in the refrigerator at -20 ℃ for spare. The qRT-PCR amplification system (total volume of 20.0 µL) consisted of: 2.0 µL of cDNA, 0.5 µL each of the upstream and downstream primers for the target gene and the internal reference gene, 10.0 µL of TB Green Premix Ex Taq II enzyme, and 7 µL of ddH_2_O, prepared as a standard reaction system. *GAPDH* was used as the internal reference, and three replicates were set up for both the target and internal reference genes. The qRT-PCR program followed a three-step protocol: 95 °C for 5 s, 60 °C for 30 s, and 72 °C for 30 s.

### Immunohistochemical staining

Immunohistochemical staining of lung tissues was primarily performed using the Streptavidin-Biotin-Peroxidase Complex (SABC) technique, with diaminobenzidine (DAB, purchased from BOSTER) used for visualization. The staining procedure was performed according to the kit instructions and included the following steps: deparaffinization of paraffin sections to water, antigen repair, blocking of endogenous peroxidase, serum blocking, addition of primary and secondary antibodies, DAB color development, restaining of cell nuclei, ethanol dehydration, and sealing of the sections with neutral balsam. Nuclei stained with hematoxylin appeared blue, and DAB-positive staining was visualized as brown. Weak or background-like brown staining was considered negative. Images were captured using a DP70 microscopic image acquisition system and analyzed under a light microscope. The mean optical density of positive areas was calculated using Image-Pro Plus 6.0 software, the calculation formula is shown in Eq. (3).

3 $$\:MOD=\frac{\sum\:{OD}_{i}}{N}$$

Where *MOD* is the mean optical density, *OD* represents the converted optical density value, calculated as $\:OD={\text{log}}_{10}(\:\frac{I}{{I}_{0}}\:)$. Here, *I* is the light intensity of the sample (corresponding to the gray value), and *I*_*0*_ is the light intensity of the background (without the sample). Additionally, *OD*_*i*_ denotes the optical density value of the i-th pixel in the region, and N is the total number of pixels in the region. These calculations are automatically performed by the Image-Pro Plus software.

The resulting data were analyzed for statistical significance using SPSS 25.0 software, with results expressed as mean ± standard deviation (X̅±SD). Data visualization was performed using Origin 2021 software (https://www.originlab.com/), with *P* < 0.05 as the criterion for the significance of differences.

## Results

### Results of cell digestion experiment

**Fig. 1:**
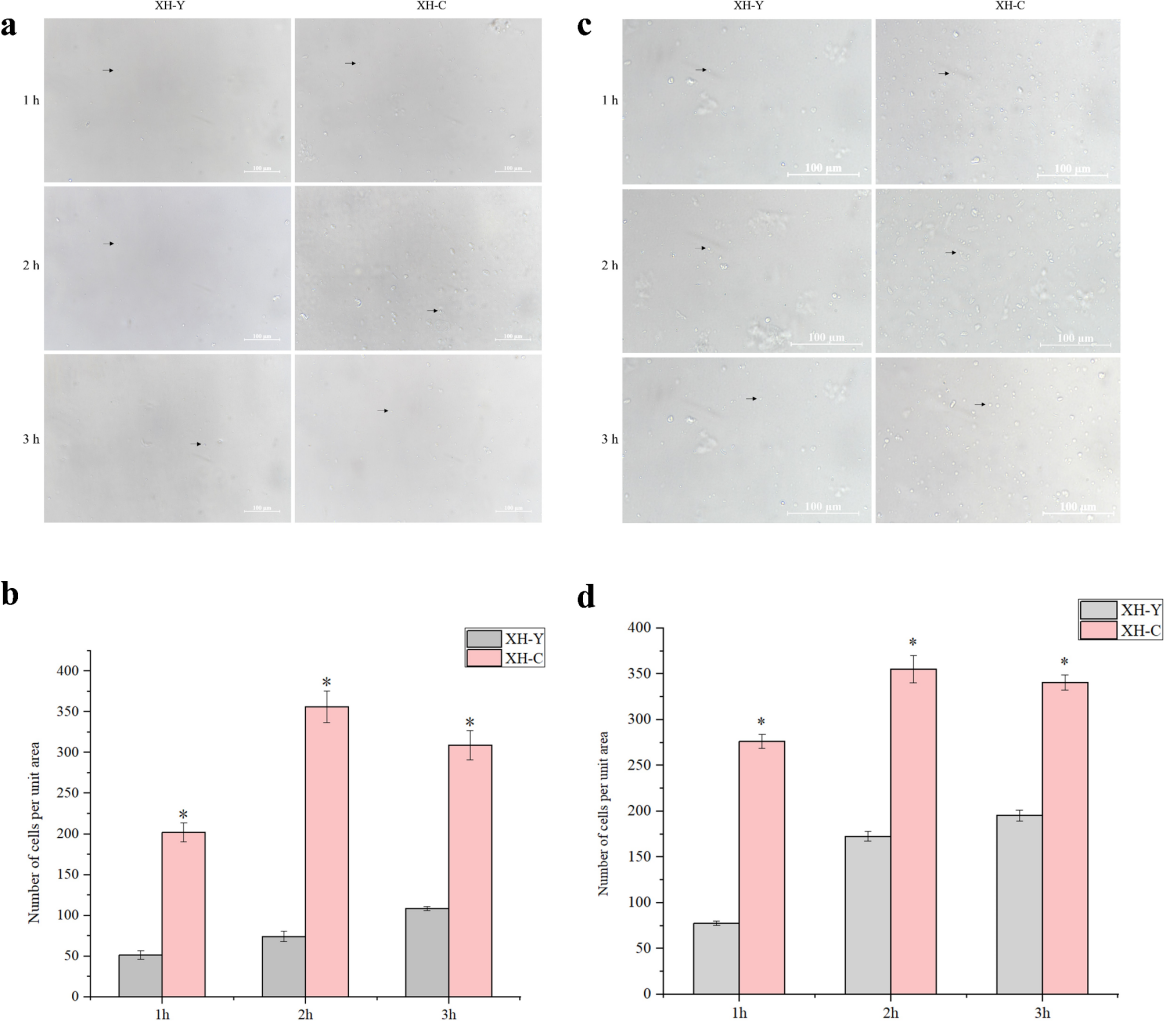
Results of digesting yaks and cattle lung tissues at the same altitude using different enzymes. (**a**) and (**b**) show the results of cell digestion and counting by trypsin. (**c**) and (**d**) show the results of cell digestion and counting by Type I collagenase. Black arrows (→) refer to the isolated cells. An asterisk (*) denotes significant difference between the two groups, *P* < 0.05.

Lung tissues from yaks and cattle at the same altitude were digested using trypsin and Type I collagenase, respectively. Digestion was terminated at 1 h, 2 h, and 3 h. After gently blowing and mixing, cells of equal size were selected for photographing and counting. Cell counting was performed using Image-Pro Plus 6.0 software, and data were analyzed using SPSS 25.0 software, with results expressed as mean ± standard deviation (X̅±SD). Visualization of the results was performed using Origin 2021 software. The results of cell digestion and counting are shown in Fig. 1. Quantitative analysis revealed that the number of cells isolated from yak lung tissues at different time points and with different enzymes was significantly lower than that from cattle (*P* < 0.05). We hypothesize that this difference arises because the connections between cells in yak lung tissues are significantly tighter, making it more difficult to separate cells during enzymatic digestion. As a result, fewer cells were isolated from yak lung tissues compared to cattle. This finding could indicate that the cell adhesion capacity of yak lung tissues is stronger than that of cattle, indicating a correlation between the number of isolated cells and cell adhesion capacity. Enhanced cell adhesion may help yaks maintain the structural stability of lung tissues and thus adapt to the plateau environment.

### GO and KEGG enrichment analysis of DEGs

**Fig. 2:**
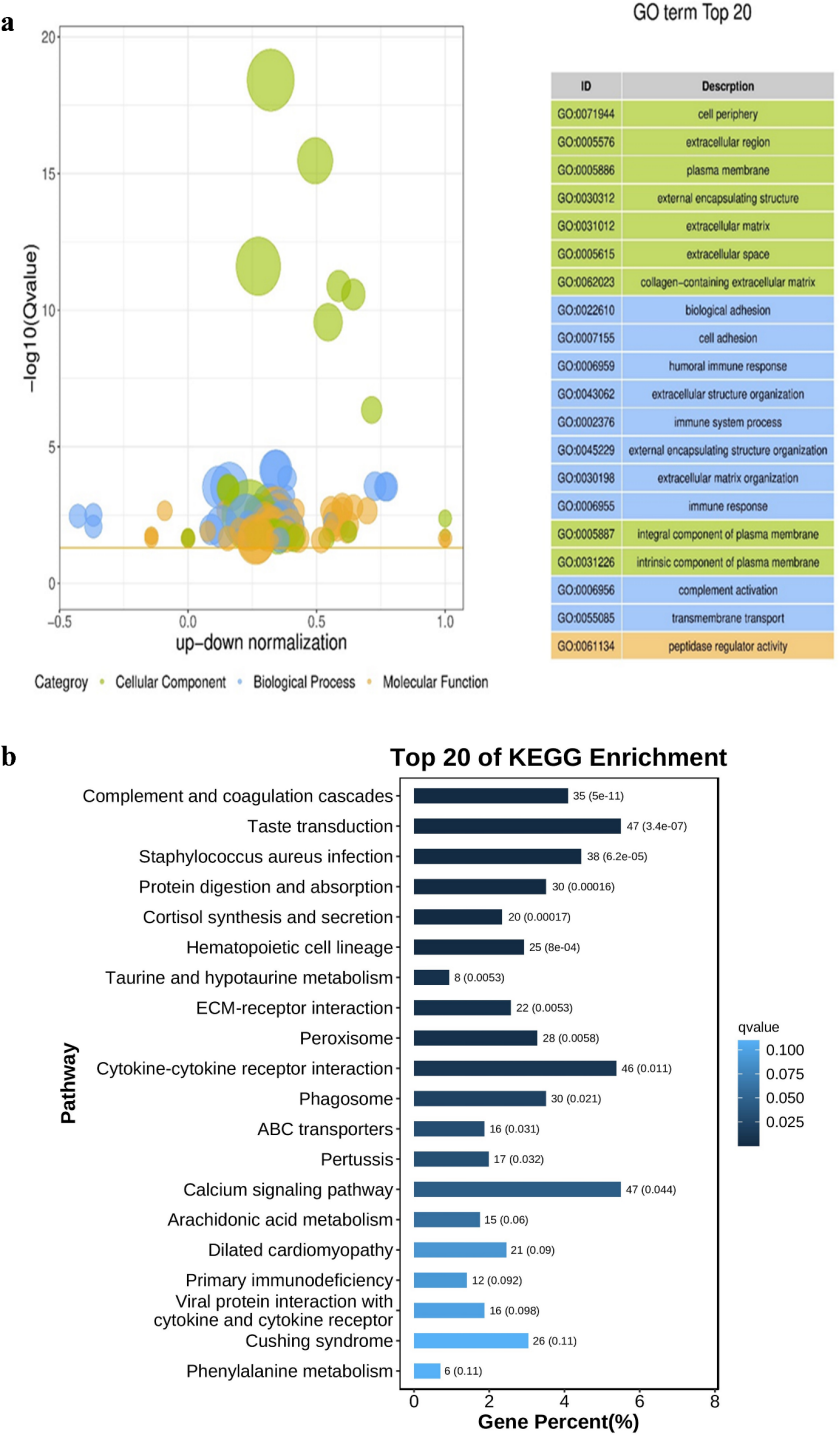
GO and KEGG enrichment analyses in lung tissues of yaks and cattle at the same altitude showed that DEGs were mainly enriched in cell adhesion-related pathways. (**a**) GO enrichment analysis of DEGs, displaying the top 20 enriched GO terms with their significance levels. The vertical coordinate is − log10 (Qvalue), the horizontal coordinate is the z-score value (the difference between the number of up-regulated differential genes and the number of down-regulated differential genes as a proportion of the total number of differential genes), and the yellow line represents the threshold of Qvalue = 0.05. (**b**) KEGG pathway enrichment analysis of DEGs highlighting the top 20 significantly enriched pathways, KEGG images are licensed from Kanehisa Laboratories (www.kegg.jp/kegg/kegg1.html). The horizontal coordinate is the ratio of the number of differential genes annotated to the total number of differential genes, the vertical coordinate is the KEGG pathway. The shade of the color represents the significance levels, the darker the color the more significant it is.

Following differential analysis of RNA-seq data from lung tissues of yaks and cattle at the same altitude, a total of 2,296 DEGs were identified in yaks compared to cattle, including 1,460 up-regulated genes and 836 down-regulated genes. Functional annotation and enrichment analysis of these DEGs were performed using the GO and KEGG Pathway databases. The results revealed that the DEGs were significantly enriched in GO terms such as biological adhesion (GO:0022610), cell adhesion (GO:0007155), and cell periphery (GO:0071944), as shown in Fig. 2a. Furthermore, the DEGs were enriched in KEGG pathways including Complement and coagulation cascades, Staphylococcus aureus infection, Hematopoietic cell lineage, ECM-receptor interaction, and Cytokine-cytokine receptor interaction. Notably, numerous DEGs were enriched in the calcium signaling pathway, which is associated with the cell adhesion process, as shown in Fig. 2b. The ECM-receptor interaction pathway is a key regulatory mechanism for cell adhesion. ECM components such as collagen and fibronectin regulate cell-matrix adhesion through interactions with cell surface receptors^30^. Genes such as *COL1A1*, *COL1A2*, *FN1*, and *ITGB4* are enriched in this pathway. *COL1A1* and *COL1A2* encode type I collagen, while *FN1* encodes fibronectin. Both proteins are essential components of the ECM, and their expression levels can influence cell adhesion capacity^31,32^. *ITGB4* mediates cell-basement membrane adhesion by binding to laminin through its extracellular domain^33^. This enhanced adhesion capacity is advantageous for yaks’ adaptation to the plateau environment.

### Measurement of cell adhesion-related ion concentrations

**Fig. 3:**
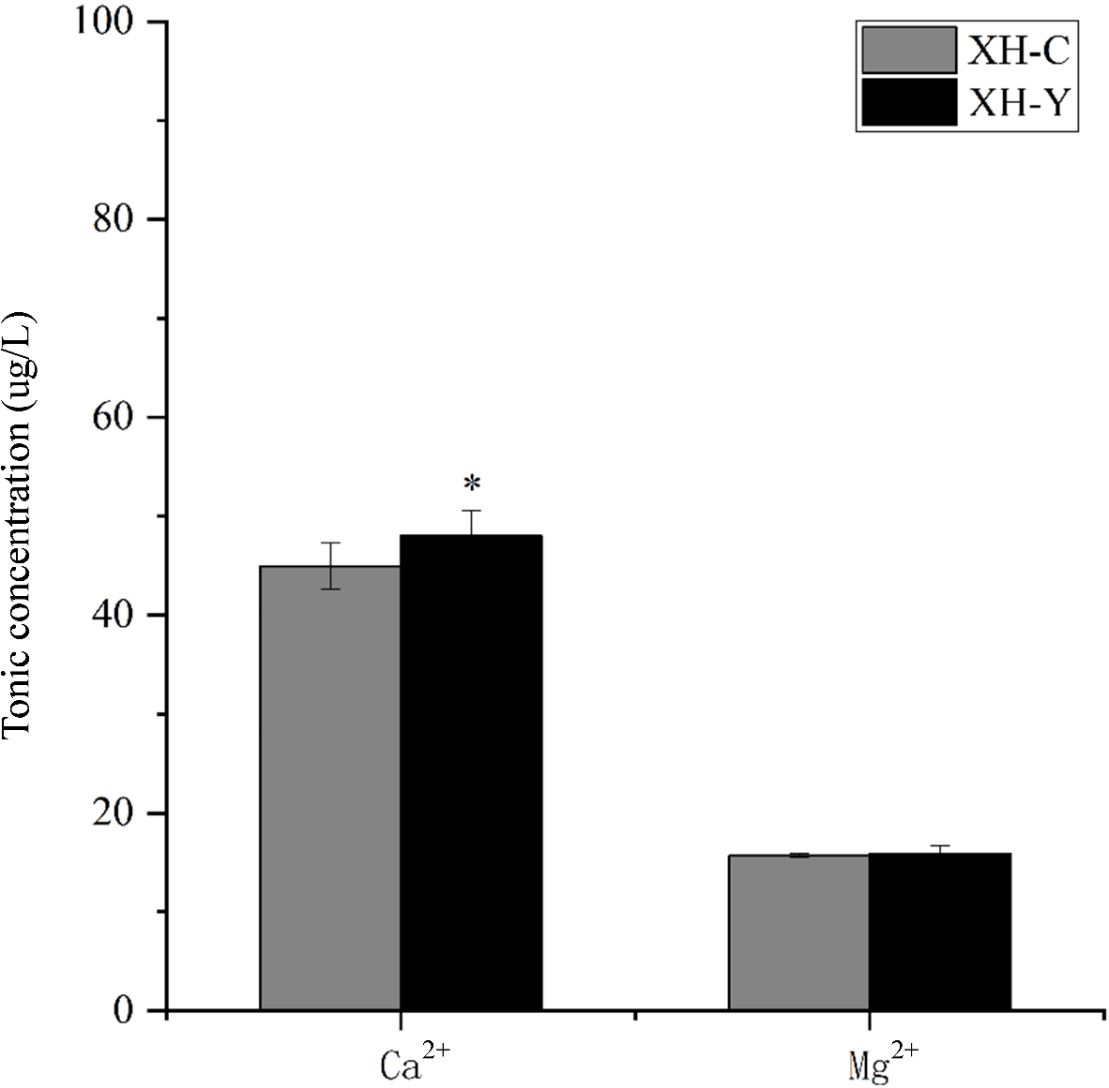
Differences in Ca^2+^ and Mg^2+^ concentrations in lung tissues of yaks and cattle.

Ca^2+^ and Mg^2+^ affect adhesion capacity by regulating integrin function, influencing signaling pathways, and organizing the cytoskeleton^34,35^. Within a certain concentration range, increased levels of both Ca^2+^ and Mg^2+^ enhance cell adhesion capacity, with their synergistic effect being particularly significant^36^. The ion concentrations in the samples were measured using ICP-MS after constant-volume dilution. The results showed that the Ca^2+^ concentration in yak lung tissues was 48.0343 µg, significantly higher than the 44.9467 µg observed in cattle at the same altitude (*P* < 0.05), whereas the Mg^2+^ concentration showed no significant change, as shown in Fig. 3. Ca²⁺ enhances the tightness of intercellular and cell-ECM interactions by binding to cadherins or activating integrin-mediated signaling pathways^37,38^. This enhanced cell adhesion is achieved through the regulation of CAMs function by elevated Ca²⁺ levels, which helps reduce tissue damage and promote tissue repair under hypoxic conditions, thereby facilitating yaks’ adaptation to the plateau environment.

### Quantitative analysis of cell adhesion-related genes

**Fig. 4:**
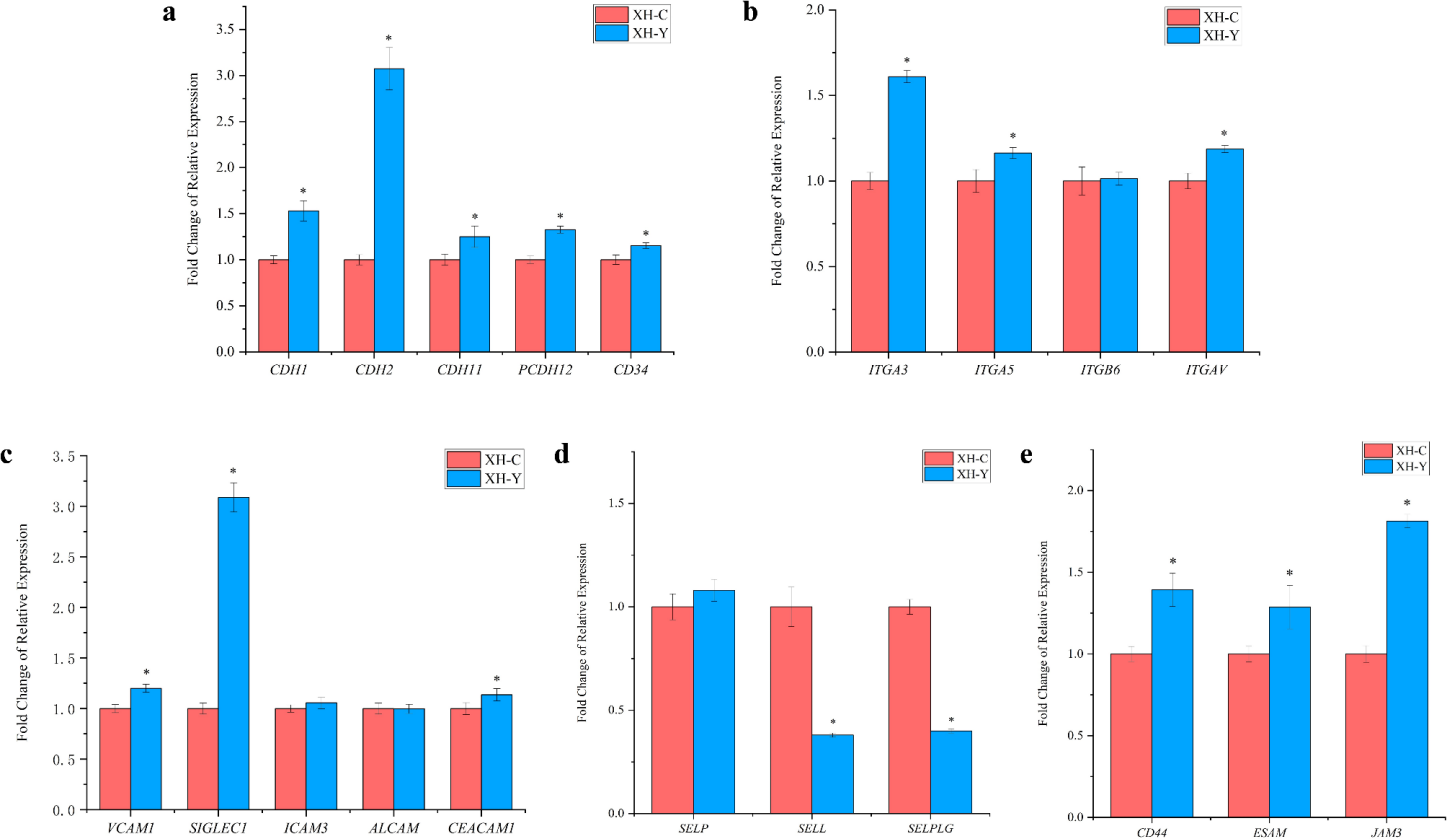
Relative mRNA expression of cell adhesion-related genes in lung tissues of yaks and cattle at the same altitude. (**a**) is cadherin family-related gene; (**b**) is integrin family-related gene; (**c**) is immunoglobulin superfamily-related gene; (**d**) is selectin family-related gene; (**e**) is other cell adhesion genes.

Following RNA extraction from lung tissues of yaks and cattle at the same altitude, qRT-PCR analysis was performed. The expression profiles of five different types of CAMs are shown in Fig. 4. In yak lung tissues, the relative expression levels of genes in the cadherin family (*CDH1*,* CDH2*,* CDH11*,* PCDH12*,* CD34*), integrin family (*ITGA3*,* ITGA5*,* ITGAV*), immunoglobulin superfamily (*VCAM1*,* SIGLEC1*,* CEACAM1*), and other cell adhesion-related genes (*CD44*,* ESAM*,* JAM3*) were significantly higher than those in cattle (*P* < 0.05). In contrast, selectin family-related genes (*SELL*,* SELPLG*) were significantly lower in yak lung tissues compared to cattle (*P* < 0.05). Among them, *CDH2* and *SIGLEC1* exhibited the highest expression levels in yak lung tissues, approximately three times higher than those in cattle. While *SELL* showed the lowest expression level in yak lung tissues, with a value of 0.3800. Notably, the expression levels of *ITGB6*,* ICAM3*,* ALCAM*, and *SELP* showed no significant differences between yaks and cattle. Among these genes, *ITGB6* mediates cell-cell and cell-ECM adhesion^39^, *ICAM3* and *ALCAM* are involved in immune cell adhesion and trafficking^40,41^, and *SELP* plays a critical role in leukocyte-endothelial cell adhesion and the regulation of inflammatory responses^42^. The lack of significant changes in the expression of these genes suggests that they are stably expressed in lung tissues. This stability indicates their potential role in maintaining fundamental cell adhesion functions and ensuring the basic functional stability of lung tissues.

### Protein expression analysis of cams

**Fig. 5:**
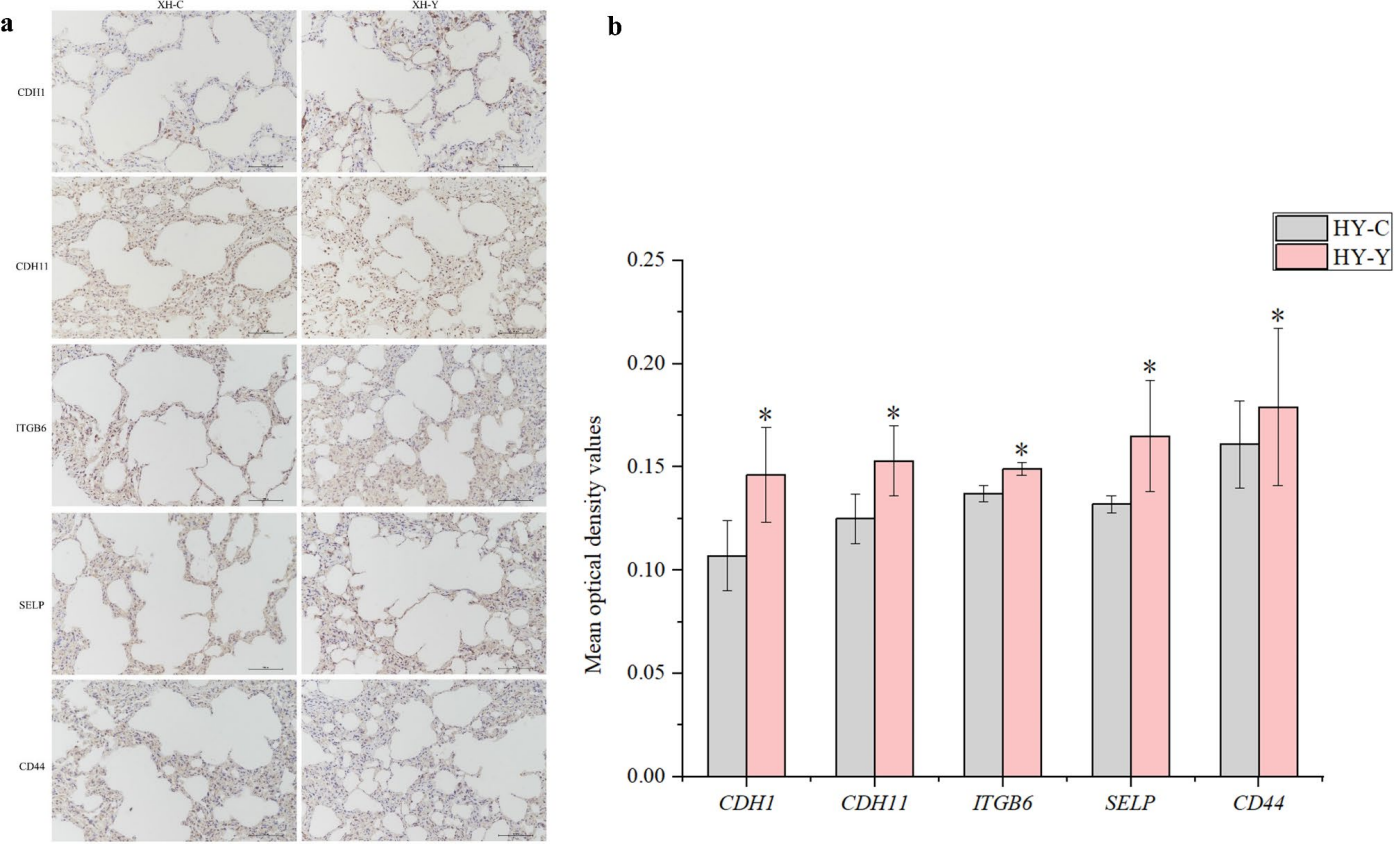
Expression levels of major adhesion molecule proteins in lung tissues of yaks and cattle analyzed by immunohistochemistry. (**a**) Immunohistochemical staining of cell adhesion-related proteins in lung tissues. (**b**) Immunohistochemical staining optical density analysis of cell adhesion-related proteins in lung tissues. An asterisk (*) denotes significant difference between the two groups, *P* < 0.05.

Following immunohistochemical staining of selected CAMs in lung tissues of yaks and cattle, we observed that the positive areas of CAMs were localized on the cell membrane surface. The mean optical density values were directly measured using Image-Pro Plus 6.0 image analysis system. The data were statistically analyzed using SPSS 25.0 software and visualized using Origin 2021 software. As shown in Fig. 5, the mean optical density values of CAM-related proteins CDH1, CDH11, ITGB6, SELP, and CD44 were significantly higher in yak lung tissues compared to cattle lung tissues at the same altitude (*P* < 0.05). Among these proteins, CD44 exhibited the highest expression in yak lung tissues, while CDH1 showed the largest expression difference between yaks and cattle, with a difference of 0.039. These findings were largely consistent with the expression trends observed in the qRT-PCR results.

## Discussions

Tissue digestion is one of the most commonly used methods for isolating primary cells from animal tissues. It primarily utilizes enzymatic properties to break down the ECM and intercellular junctions, thereby separating individual cells from the tissue structure. Collagenase and trypsin are frequently employed for cell separation experiments. Collagenase acts relatively gently and can specifically hydrolyze the three-dimensional helical structure of native collagen fibers, it can be classified into multiple types based on differences in specificity, with Type I, Type II, and Type III being common^43^. Trypsin possesses unique proteolytic capabilities, enabling precise degradation of protein components within the ECM, thereby promoting effective cell dissociation^44^. Our study observed that regardless of whether trypsin or Type I collagenase was used to digest lung tissue, the number of cells isolated per unit area at 1 h, 2 h, and 3 h was significantly lower in yaks compared to cattle. It can be seen that the cell adhesion capacity in lung tissues differs between yaks and cattle, with yak lung tissues demonstrating stronger intercellular or cell-to-matrix adhesion compared to cattle.

Cell adhesion is a process in which cells attach to each other or to the underlying matrix (e.g., the ECM) through CAMs, playing a central role in cell migration^45^. The adhesive capacity between cells is critical for determining cell polarity and is essential for maintaining the morphology of tissues composed of cells^46^. Most CAMs are Ca^2+^-dependent proteins, with calmodulin binding to Ca^2+^, and the more Ca^2+^ is bound, the more rigid the CAMs become^47^. Many CAMs rely on Ca^2+^ to function, and in the presence of Ca²⁺, they promote cell adhesion and resist hydrolysis by proteases^48^. Changes in intracellular Ca^2+^ levels can modulate the expression of immunoglobulin superfamily, cadherin family, integrin family, and other CAMs^49^. In this study, the Ca^2+^ content in yak lung tissues was significantly higher than that in cattle. It can be inferred that Ca^2+^ concentration is associated with the expression of CAMs in yak lung tissues, and yaks exhibit stronger intercellular adhesion within lung tissue compared to cattle.

RNA-seq results revealed that DEGs were significantly enriched in cell adhesion-related pathways, mainly in related GO and KEGG pathways including cell adhesion (GO:0007155), CAMs, and Calcium signaling pathway. qRT-PCR and immunohistochemical analyses further identified changes in the expression of five different types of CAMs, with the expression levels of key CAM-related genes and proteins being significantly higher in yaks compared to cattle. The classical cadherin genes, including *CDH1*,* CDH2*, and *CDH11*, are critical intercellular adhesion molecules that regulate cell movement, clustering, and differentiation, thereby promoting morphogenesis and tissue barrier formation^50^. These cadherin-related genes play a vital role in regulating lung inflammation and maintaining the alveolar-capillary barrier in yaks. Genes from the integrin family, such as *ITGA3*,* ITGA5*, and *ITGAV*, are involved in endothelial cell migration, cytoskeletal organization, apoptosis initiation, and the regulation of lung inflammation and fibrosis^51,52^. Members of the immunoglobulin superfamily, including *VCAM1*,* SIGLEC1*, and *CEACAM1*, participate in interactions between cells and pathogens to promote effective phagocytosis and antigen presentation of adaptive immune response, and related genes play a role in regulating the body’s immune functions^53–56^. *SELP* and *SELPLG* belong to the selectin family. *SELP* triggers the release of procoagulant microparticles and increases the expression of tissue factor on monocytes, playing a pivotal role in hemostasis and leukocyte recruitment^57^. *SELPLG* is essential for leukocyte trafficking during inflammation^58^. Other CAMs, such as *CD44*,* ESAM*, and *JAM3*, are involved in angiogenesis, leukocyte transendothelial migration, and platelet activation^59–61^. It can be seen that the high expression of CAMs in yak lung tissues likely contributes to their enhanced adaptation to the plateau environment.

In summary, the expression levels of genes and proteins related to cell adhesion were significantly higher in yak lung tissues compared to those cattle. We hypothesize that this enhanced expression in yak lung tissues may strengthen immune responses, improve lung tissue repair capacity, and maintain the structural integrity of the air-blood exchange barrier. Additionally, these molecules may contribute to intrapulmonary angiogenesis, thereby facilitating better adaptation to the hypoxic conditions of the plateau environment. However, this study has several limitations. Due to the challenges associated with obtaining tissue samples from large animals in their natural habitats, we were restricted to a minimal biological sample size for our analysis. This small sample size may limit the generalizability and statistical significance of our findings, making it difficult to fully capture the differences in cell adhesion among different species. Furthermore, the application of our method is not comprehensive and lacks further functional experimental validation. We will increase the sample size in subsequent studies to comprehensively analyze the differences in the expression of CAMs in lung tissues under different conditions and validate the findings with functional experiments to elucidate the relationship between CAMs and adaptation to the plateau environment. Although this study reveals one aspect of plateau adaptation in yaks, further work is needed to refine and extend these findings.

## Conclusions

The present study demonstrates that cell adhesion, cell adhesion-related genes and proteins in yak lung tissue were significantly higher than those in cattle. These findings suggest that yaks may enhance the immune effects of lung tissue repair by increasing adhesion ability and upregulating the expression of CAMs, maintaining the integrity of the lung endothelial barrier, and promoting angiogenesis within the lungs, thereby better adapting to the hypoxic conditions of the plateau environment. In the future, the conclusions drawn from this study could be applied to other fields. By elucidating the mechanisms of cell adhesion in yaks’ adaptation to hypoxic environments may provide valuable insights for developing conservation strategies for other plateau-dwelling animals. Genes and proteins associated with cell adhesion in yaks could serve as potential therapeutic targets, offering new avenues for the development of drugs to treat high-altitude-related diseases.

## Data Availability

Sequence data that support the findings of this study are available in the NCBI SRA with the primary accession code PRJNA1127767.
